# *Buxus natalensis* (Oliv.) Hutch (Buxaceae) Exhibits Its Anticancer Potential by Stimulating ROS Production and Caspase-p53-BCL-2-Dependent Apoptosis in Hepatocellular Carcinoma and Prostate Cancer Cell Lines

**DOI:** 10.3390/ijms26094173

**Published:** 2025-04-28

**Authors:** Emmanuel Mfotie Njoya, Gaetan T. Tabakam, Chika I. Chukwuma, Tshepiso J. Makhafola

**Affiliations:** Centre for Quality of Health and Living, Faculty of Health and Environmental Sciences, Central University of Technology, Bloemfontein 9301, Free State, South Africa; tgaetan@cut.ac.za (G.T.T.); cchukwuma@cut.ac.za (C.I.C.)

**Keywords:** apoptosis, BAX/BCL-2 ratio, ROS, caspases, *Buxus natalensis*, phytochemicals, cancer

## Abstract

*Buxus natalensis* is recognized as a rich source of triterpenoidal alkaloids that are known to be effective in fighting different cancer types. Nevertheless, to date, no anticancer potential of *B. natalensis* extract has been yet described. Here, we investigated the antiproliferative activity of different *B. natalensis* leaf extracts on eight cancer cell lines (MCF-7, 4T1, Caco-2, HeLa, A549, HepG2, DU145, and LNCaP). Chang liver cell line derived from normal liver tissue, was used as control. *B. natalensis* hydroethanolic leaf extract (BNHLE) was found to exert significant cytotoxic effect against cancerous cell lines, with the highest efficacy being observed on LNCaP and HepG2 with IC_50_ values of 47.39 and 78.01 µg/mL, respectively. Interestingly, BNHLE was less cytotoxic towards Chang liver cells with an IC_50_ value of 334.10 µg/mL, yielding selectivity index (SI) values of 6.96 and 4.22 against LNCaP and HepG2 cells, respectively. The study of mechanism of action revealed that BNHLE exerted its antiproliferative effect by inducing ROS production and caspase -3/-7, and -9 activities in LNCaP and HepG2 cells. Moreover, it was found that BNHLE activated apoptosis in both cancerous cell lines by enhancing the expression levels of p53, while suppressing the expression of NF-κB-p65 and BCL-2 protein levels in a dose-dependent manner. The phytochemical analysis of BNHLE showed the presence of flavonoids (24.45 mgQE/g extract) and phenolics (84.64 mgGAE/g extract), and its LC-MS profiling identified several compounds including robinin and rutin, which are known for their cytotoxic effect against different cancer cell lines, such as hepatocellular carcinoma and prostate cancer cell lines. Several compounds are still unknown from *B. natalensis*, but the data obtained so far justify the use of *B. natalensis* as a potential source of bioactive compounds against hepatocellular and prostate cancers.

## 1. Introduction

Cancer is a group of diseases caused by various risk factors that induce genetic and epigenetic changes leading to abnormal and uncontrolled cell division that can invade adjoining parts of the body and spread to other organs [1,2]. It is one of the leading causes of morbidity and mortality worldwide, with approximately 20 million new cases and nearly 10 million cancer-related deaths in 2022 [3]. Breast, lung, colorectal, prostate, skin, and stomach cancers are reported as the most common cancers worldwide, with lung and colorectal cancers having the highest mortality rates [4]. In South Africa, almost 110,000 new cases of cancer were diagnosed with more than 56,000 deaths, and the incidence of new cases is expected to rise to 138,000 and 175,000 by 2030 and 2040, respectively [5]. Breast, cervical, basal cell carcinoma, squamous cell carcinoma, and colorectal cancers are the most commonly diagnosed in South African women, while prostate, basal cell carcinoma, squamous cell carcinoma, colorectal, and lung cancers are mostly diagnosed in men [6]. Overall, the five most diagnosed cancers in both sexes are breast, prostate, cervical, lung, and colorectal cancers [5]. Today, several cancer types can be prevented via the control and monitoring of known risk factors. Oxidative stress and chronic inflammation are among the key risk factors that promote most stages of cancer initiation and development, and several studies have reported their implication in the pathogenesis of various malignancies [7,8,9,10]. Further, many studies suggested that reactive oxygen species (ROS) play a dual role in cellular metabolism [7,11,12]. At low to moderate levels, ROS can act as signaling molecules that promote cell proliferation, migration, invasion, and angiogenesis, contributing to tumour growth, while high levels of ROS can induce damage to macromolecules such as DNA, proteins, and lipids, thus enhancing cancer cell death [13,14]. Additionally, numerous studies have proved that cancer cells have high levels of ROS compared to normal cells, and most chemotherapeutics increase intracellular levels of ROS to weaken the antioxidant system of cancer cells, thus promoting apoptotic cell death [15,16,17]. Besides, the activation of inflammatory cells during oxidative stress can generate growth factors including cytokines that promote the development of tumor cells [18]. The accumulation of such events over time is the cornerstone for cancer initiation and development. Therefore, targeting oxidative stress or inflammatory conditions represents an effective strategic approach for both cancer prevention and therapy.

The treatment of cancer includes immunotherapy, chemotherapy, radiotherapy, stem cell therapy, and chirurgical intervention used either as a single treatment or in combination [19,20]. Despite their cost and side effects, which impact the quality of life of treated patients, these treatment methods do not always give satisfactory results. In addition, drug resistance and the failure to clear all the tumour cells lead to the proliferation of surviving cancer cells. Therefore, it is urgent to search for alternative and effective anticancer drugs. Scientific reports have indicated the importance of medicinal plants as sources of phytochemicals with anticancer potential that could be used for cancer drug development [21,22,23]. Phytochemicals exhibit anticancer efficacy via diverse modes of action. One of these mechanisms is chemoprevention, during which phytochemicals exert their anticancer activity owing to their antioxidant potential or by blocking and suppressing carcinogens that might induce DNA mutation to stimulate cancer initiation [24]. Other phytochemicals exhibit their anticancer potential by inducing ROS-mediated cytotoxicity in cancer cells, or activate cancer cell death pathways such as apoptosis and autophagy in association with cell cycle arrest, inhibition of cell invasion and migration, or by modulating cell signalling pathways that promote cancer development [25,26].

Plants from the genus Buxus are used in folk medicine to treat various illnesses. Pharmacological and phytochemical analyses of these plants have highlighted the presence of more than 200 steroidal alkaloids [27,28], with the evidence that some of these alkaloids exhibited promising bioactivities including anticancer [29,30,31], antiprotozoal [32,33], immunosuppressive [34], and acetylcholinesterase inhibitory activities [35]. *Buxus natalensis* is an attractive garden plant that is used to treat memory loss in elderly people [35], and the phytochemical analysis has revealed that it contains several triterpenoidal alkaloids that can be effective in fighting different cancer types. However, to the best of our knowledge, no anticancer potential of *B. natalensis* extract has been yet reported. Therefore, the current study aims to evaluate the antiproliferative effect of different *B. natalensis* leaf extracts, and further investigates the mechanistic pathways affected by the bioactive extract on the most sensitive cancer cells. Additionally, a phytochemical analysis was conducted to determine the compounds that might be responsible for the antiproliferative property of this plant species.

## 2. Results and Discussion

### 2.1. Buxus natalensis Leaf Extracts Trigger Differential Cytotoxic Effect Between Cancer Cell Lines and Normal Cell Line

**Figure 1:**
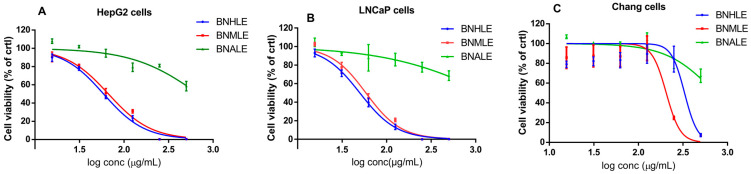
Cytotoxic effect of *Buxus natalensis* leaf extracts on HepG2 (**A**), LNCaP (**B**), and Chang liver (**C**). Ten thousand cells were seeded per well for each cell line on 96-well microtiter plates, and the cells were treated for 48 h with different leaf extracts (15.625–500 µg/mL) under standard cell-culture conditions. The cell viability was estimated as a percentage of cells treated with DMSO (0.5%) considered as 100%. *B. natalensis* hydroethanolic leaf extract (BNHLE); *B. natalensis* methanolic extract (BNMLE); *B. natalensis* aqueous leaf extract (BNALE).

The search for safe and effective anticancer treatments is still a top priority, especially in developing nations where the incidence of cancer is disproportionately high due to a lack of adequate healthcare resources [36]. For this reason, the investigation of plant-derived substances appears as an alternative strategy to discover natural compounds that may be useful for the development of novel anticancer agents. Therefore, the cytotoxic effect of *B. natalensis* leaf extracts obtained from three solvent systems was assessed for 48 h on eight cancer cell lines and compared to a normal cell line. The cell viability was estimated as a percentage of cells treated with DMSO (0.5%). In fact, preliminary work was done to confirm DMSO concentration which is non-cytotoxic to each cell line, and it is generally known that DMSO at 0.5% is considered safe for most cell cultures [37]. According to the criteria of the National Cancer Institute [38], our results showed that *B. natalensis* leaf extracts have low cytotoxic effect against A549, HeLa, 4T1, MCF-7, DU145, and Caco-2 cell lines (Figure S1, Table 1) with IC_50_ values greater than 100 µg/mL. In contrast, *B. natalensis* hydroethanolic leaf extract (BNHLE) and methanolic extract (BNMLE) inhibited the growth of HepG2, LNCaP cells in a concentration-dependent manner, while a less cytotoxic effect was observed with *B. natalensis* aqueous leaf extract (BNALE) (Figure 1). Based on the classification of the National Cancer Institute, BNHLE and BNMLE exhibited moderate efficacy (30 μg/mL < IC_50_ < 100 μg/mL) towards both cancer cells (HepG2, LNCaP) compared to normal cells (Chang liver), thereby suggesting the presence of cytotoxic compounds in these plant extracts, which selectively kill cancer cell types with minimal toxic effect against a normal cell line. The selective cytotoxic effect of these plant extracts is advantageous in cancer therapy, and this selectivity is mainly attributed to the difference between cancer and normal cells in terms of variations in cell cycle regulation, DNA repair capabilities, or the presence of unique cancer-specific targets [39]. Thus, BNHLE and BNMLE are potential sources of safe and effective compounds against hepatocellular and prostate cancer cells. This finding is consistent with other reports indicating a concentration-dependent antiproliferative effect of the hydroethanolic extract of *Buxus sempervirens*, a plant of Buxaceae family, which inhibited the growth of human cancer cell lines, including BMel melanoma, HCT116 colorectal, and PC3 prostate cancer cells [29].

Table 1 summarizes the inhibitory concentrations (IC_50_) values determined using a non-linear regression curve of percentage of cell viability versus the logarithm (log10) of concentrations of each sample. From this table, it resulted that BNALE was not effective against all cell lines up to 500 µg/mL. Besides, BNHLE and BNMLE exerted similar cytotoxic effect against cancerous cells, with the highest efficacy being observed on HepG2 cells with IC_50_ values of 78.01 and 79.07 µg/mL, and LNCaP with IC_50_ values of 47.39 and 49.18 µg/mL, respectively. Interestingly, BNHLE was less cytotoxic towards Chang liver cells with an IC_50_ value of 329.70 µg/mL yielding selectivity index (SI) values of 6.96 and 4.22 against LNCaP and HepG2 cells, respectively (Table 2). In the same way, BNMLE was more cytotoxic against Chang liver cells with an IC_50_ value of 205.50 µg/mL, yielding selectivity index (SI) values of 4.17 and 2.59 against LNCaP and HepG2 cells, respectively. On the contrary, doxorubicin, used as the most widely chemotherapeutic drug against many cancer types [40], showed poor SI values of 0.29 and 0.71 against HepG2 and LNCaP cells. Selectivity index is a reliable parameter that determines the capacity of plant extract or a given substance to inhibit the growth of malignant cells while preserving healthy, normal cells [41,42]. Our study showed that BNHLE and BNMLE have higher selectivity against LNCaP and HepG2 cells, compared to a poor selectivity with doxorubicin, and this emphasizes the presence of potent phytochemicals targeting specific functions of malignant cells, thus inhibiting their growth and survival. Moreover, antioxidant compounds within these plant extracts may undoubtedly prevent oxidative damage on normal cell lines. Therefore, our data suggest that BNHLE and BNMLE are better drug candidates for growth inhibition of both cancer cell lines based on their SI values greater than 1. Additionally, knowing that an SI greater than 1 indicates a higher cytotoxic effect against cancer cells than against normal cells, and the greater the SI value is, the more selective it is [43,44], we decided to focus our subsequent experiments on BNHLE, which showed the highest SI values compared to BNMLE.

**Figure 2:**
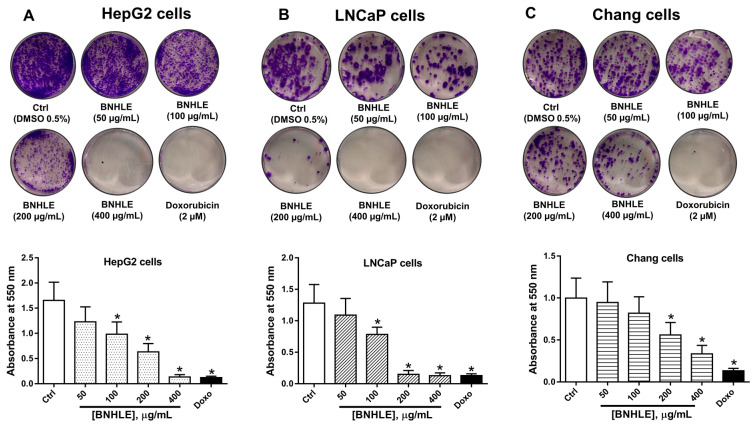
Colony formation after treatment with *B. natalensis* hydroethanolic leaf extract (BNHLE). One thousand cells from each cell line HepG2 (**A**), LNCaP (**B**), and Chang liver (**C**) were seeded in 6-well microtiter plates, and exposed to BNHLE for 24 h, followed by the replacement of the culture medium every 3 days for 15 days. The colonies were stained with crystal violet (0.5%) and allowed to dry overnight. Then, 2 mL of acetic acid (33%) was added per well, and the absorbance was read at 550 nm using a SpectraMax iD3 multi-mode microplate reader (Molecular Devices, San Jose, CA, USA). One-way ANOVA combined with Dunnett or Student–Newman–Keuls’s tests were used for data analysis. * means statistically different (*p* < 0.05) vs. Ctrl.

To further assess the cytotoxic effect of the prominent extract (BNHLE) against the most sensitive cancer and normal cells, we evaluated its antiproliferative potential using the colony formation assay, which measures the ability of a single cell to grow into a colony after exposure to a substance [45]. Therefore, both cancer cells (HepG2 and LNCaP) and normal Chang liver cells were treated with different concentrations of BNHLE for 24 h, and the grown colonies were quantified after 15 days as indicated in Figure 2.

It was found that BNHLE at 100, 200, and 400 µg/mL, as well as doxorubicin (2 µM), induced significant damage to HepG2 and LNCaP cells, which resulted in growth suppression and significant reduction in the formation of colonies (Figure 2A,B). Additionally, LNCaP cells were highly sensitive to BNHLE as only few colonies were observed at 200 µg/mL, and no colony at 400 µg/mL (Figure 2B). However, Chang liver cells were more resistant to BNHLE, and could only induce a significant reduction on the formation of colonies at 200 and 400 µg/mL (Figure 2C). These data supported the antiproliferative potential of BNHLE against HepG2 and LNCaP cells with less cytotoxic effect against normal Chang liver cells. Thus, BNHLE can be considered as a source of potential anticancer phytochemicals that selectively kill cancer cells with minimal cytotoxic effect on normal cells.

### 2.2. Buxus natalensis Hydroethanolic Leaf Extract Induces the Intracellular Production of Reactive Oxygen Species in Cancer Cells

Reactive oxygen species (ROS) constitute a group of highly reactive molecules that play a dual role in cellular metabolism. As such, at low to moderate levels, ROS can disrupt cellular processes, including cell proliferation, migration, invasion, and angiogenesis, contributing to tumour growth, while high levels of ROS can induce damage to macromolecules such as DNA, proteins, and lipids, leading to cellular dysfunction or cell death [13,14]. Additionally, numerous studies have proved that cancer cells have high levels of ROS compared to normal cells, and most chemotherapeutics increase intracellular levels of ROS to weaken the antioxidant system of cancer cells, thus promoting apoptotic cell death [15,16,17]. Therefore, the quantification of intracellular levels of ROS could be a promising strategy to assess the anticancer potential of drug substances.

**Figure 3:**
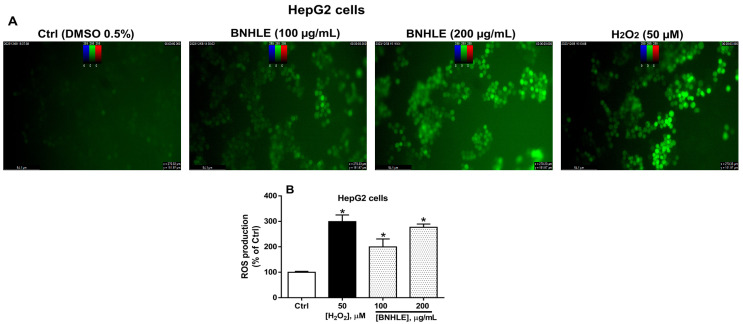
Intracellular ROS production in HepG2 cells after treatment with *B. natalensis* hydroethanolic leaf extract (BNHLE). HepG2 cells (10,000 cells/well) were treated for 45 min with 100 µL of 20 µM of DCFH-DA diluted in culture medium containing 2% FBS. Thereafter, these cells were exposed for 24 h to BNHLE (100, and 200 µg/mL), and hydrogen peroxide (H_2_O_2_, 50 µM) or DMSO (0.5%) used as negative control (Ctrl). Images were captured using a Flexacam C1 camera (Leica Microsystems GmbH, Wetzlar, Germany) connected to a fluorescence microscope (Leica Microsystems GmbH, Wetzlar, Germany) using an excitation/emission filter (480/535 nm) on 20× objective (**A**). The cell fluorescence was measured at the wavelengths of 485 nm (excitation) and 535 nm (emission) on a SpectraMax iD3 multi-mode microplate reader (Molecular Devices, San Jose, CA, USA), and intracellular ROS levels were expressed as percentage of negative control cells (**B**). One-way ANOVA combined with Dunnett or Student–Newman–Keuls’s tests were used for data analysis. * means statistically different (*p* < 0.05) vs. Ctrl.

**Figure 4:**
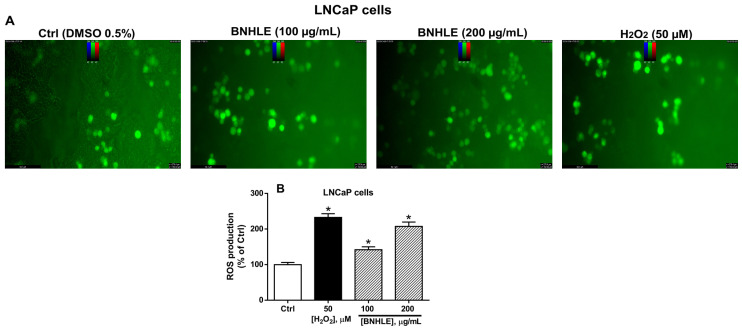
Intracellular ROS production in LNCaP cells after treatment with *B. natalensis* hydroethanolic leaf extract (BNHLE). LNCaP cells (10,000 cells/well) were treated for 45 min with 100 µL of 20 µM of DCFH-DA diluted in culture medium containing 2% FBS. Thereafter, these cells were exposed for 24 h to BNHLE (100, and 200 µg/mL), and hydrogen peroxide (H_2_O_2_, 50 µM) or DMSO (0.5%) used as negative control (Ctrl). Images were captured using a Flexacam C1 camera (Leica Microsystems GmbH, Wetzlar, Germany) connected to a fluorescence microscope (Leica Microsystems GmbH, Wetzlar, Germany) using an excitation/emission filter (480/535 nm) on 20× objective (**A**). The cell fluorescence was measured at the wavelengths of 485 nm (excitation) and 535 nm (emission) on a SpectraMax iD3 multi-mode microplate reader (Molecular Devices, San Jose, CA, USA), and intracellular ROS levels were expressed as percentage of negative control cells (**B**). One-way ANOVA combined with Dunnett or Student–Newman–Keuls’s tests were used for data analysis. * means statistically different (*p* < 0.05) vs. Ctrl.

Figure 3 and Figure 4 describe the intracellular ROS production in HepG2 and LNCaP cells after treatment with BNHLE, respectively. As presented in these figures, low ROS production is observed in cancer cells treated with 0.5% DMSO (Ctrl). However, a concentration-dependent and significant increase of the intracellular levels of ROS are observed after exposure of both cancer cells to BNHLE (100 and 200 µg/mL) as compared to control cells. Similarly, hydrogen peroxide (H_2_O_2_, 50 µM) used as positive control also caused a significant increase of ROS production in cancer cells. Other findings reported that several plant extracts can induce anticancer activity partly by stimulating the generation of ROS within cancer cells [46,47,48]. The ROS generation by BNHLE imply the activation of oxidative stress, which can disrupt cellular processes and ultimately lead to cancer cell death. This is mainly due to the fact that cancer cells are susceptible to higher levels of ROS, unlike normal cells, and this high level of ROS ultimately decreases the antioxidant capacity of cancer cells and results in selective targeting and apoptosis [49]. This difference has been exploited therapeutically to yield the selective cytotoxic effect of BNHLE. Based on our results, it can be suggested that BNHLE contains effective phytochemicals that cause growth inhibition or cell death by promoting ROS-induced apoptosis in HepG2 and LNCaP cancer cells.

### 2.3. Buxus natalensis Hydroethanolic Leaf Extract Increases the Caspase Activity in Cancer Cells

Caspases are a family of proteases that are crucial in both initiation and execution of the apoptotic process. In fact, initiator caspases-2, -8, -9, and -10 are involved in apoptosis induction while executioner caspases-3, -6, and -7 are implicated in apoptosis execution [50,51]. Thus, targeting caspases is a promising avenue for cancer therapy. In this regard, we further explored the effect of BNHLE on the induction of apoptosis in HepG2 and LNCaP cells using commercially available kits.

**Figure 5:**
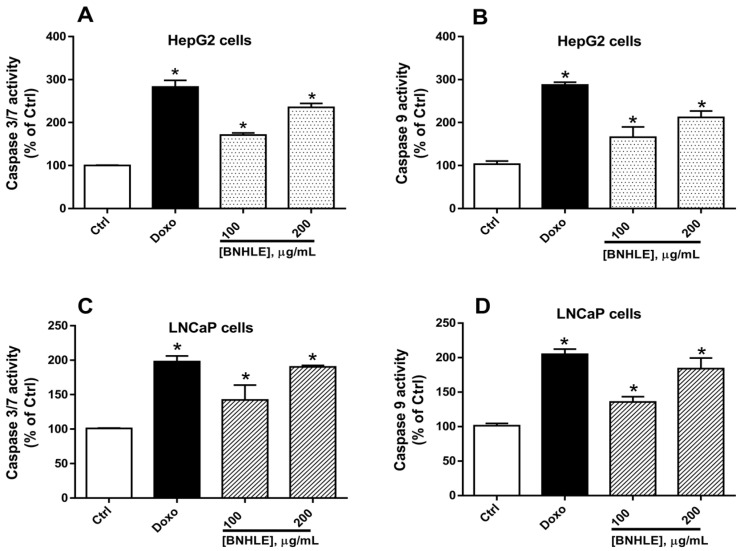
Caspase luminescence activity in HepG2 (**A**,**B**) and LNCaP cells (**C**,**D**) after exposure to *B. natalensis* hydroethanolic leaf extract (BNHLE). HepG2 and LNCaP cells (10,000 cells/well) were exposed for 18 h to BNHLE and doxorubicin hydrochloride (Doxo, 2 µM) or DMSO (0.5%) used as negative control (Ctrl). Caspase reagents were added in equal volume as the culture medium, and luminescence was read using SpectraMax iD3 multi-mode microplate reader (Molecular Devices, San Jose, CA, USA). Caspase activities were expressed as a percentage (fold change) of cells treated with DMSO (0.5%) used as a negative control. One-way ANOVA combined with Dunnett or Student–Newman–Keuls’s tests were used for data analysis. * means statistically significantly different (*p* < 0.05) vs. Ctrl.

As presented in Figure 5, our results showed that BNHLE induced a statistically significant (*p* < 0.05) and concentration-dependent activation of the expression of caspases-3/-7, and -9 in HepG2 and LNCaP cells with the highest fold change reaching 1.5 to 2 as compared to control cells. In fact, two different mechanisms may explain the induction of apoptosis via the activation of caspases: initiator caspases may convert various signals into protease activity and apoptosis arises via the activation of either death-inducing signaling complexes (caspase-8) or apoptosome (caspase-9); in another way, executioner caspases (caspase -3 and -7) may cleave various cytoplasmic or nuclear substrates inducing the occurrence of morphologic characteristics of apoptosis [52,53]. Another study reported the caspase 3-dependent apoptosis by the acetone extract of *Buxus sempervirens* in BT-20 and T47D breast cancer cells [30]. As it was found that BNHLE induced a greater increase of both initiator and executioner caspases in HepG2 and LNCaP cells, it can be suggested that BNHLE promoted apoptosis by activating the expression of an initiator caspase (such as caspase-9), which in turn stimulates an executioner caspase (such as caspase-3 or -7), leading to morphological changes that finally resulted in the growth inhibition or programmed cell death of HepG2 and LNCaP cells. A similar finding was suggested with the acetone extract of *Buxus sempervirens,* which triggers apoptosis by activating the expression of caspase 9 that activates executor caspases (caspases -3 and -7) [30]. On the other hand, different time points were used according to the endpoint of each experiment, and this is due to the fact that the effectiveness of drugs and their underlying mechanisms on cancer cells can vary significantly over time. More precisely, different time points in various experiments are used to observe changes in cell viability, tumor growth, and specific molecular markers of drug efficacy in cells. For instance, in cytotoxicity assay, 48 h of cell treatment is suitable to assess if a sample induced cell death, while 18–24 h of treatment is required to activate apoptotic markers in cancer cells, and these time points are selected based on preliminary data from our previous work [44,54].

### 2.4. Buxus natalensis Hydroethanolic Leaf Extract Modulates the Expression Levels of Human p53, BCL2, BAX, and NF-κB-p65 in Cancer Cells

**Figure 6:**
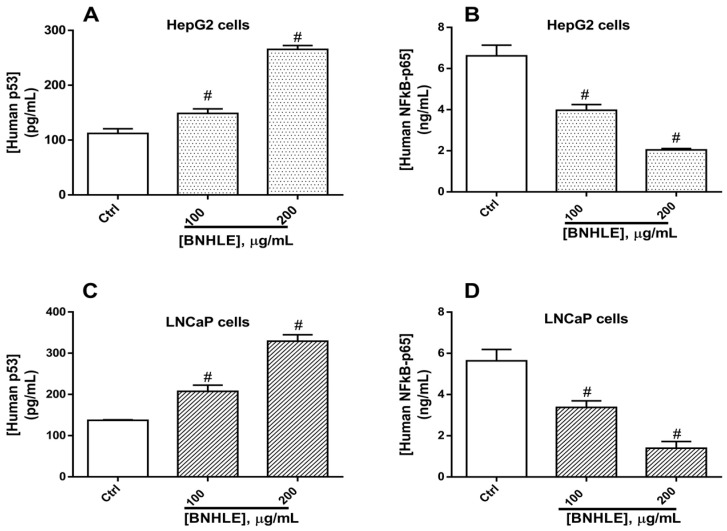
Expression levels of human p53 and NF-κB-p65 in HepG2 (**A**,**B**) and LNCaP (**C**,**D**) after 24 h of treatment with *B. natalensis* hydroethanolic leaf extract (BNHLE). Cells were treated for 24 h with BNHLE, or DMSO (0.5%) used as negative control (Ctrl). Equal amounts of proteins in cell lysates were used for the quantification of the expression levels of NF-κB-p65 and p53 using commercially available ELISA kits (Cat# E-EL-H1388 and Cat# E-EL-H0910, Elabscience Biotechnology Inc., Houston, TX, USA). One-way ANOVA combined with Dunnett or Student–Newman–Keuls’s tests were used for data analysis. # means statistically significantly different (*p* < 0.05) vs. Ctrl.

Numerous studies have shown the importance of the tumour suppressor p53 in the prevention of tumour development, and this is mainly due to its central role as a negative regulator of cell growth through induction of apoptosis and/or cell cycle arrest [55,56]. In fact, p53 is a transcription factor, which acts by activating the expression of pro-apoptotic genes (BAX) and suppressing anti-apoptotic genes (BCL-2), thereby triggering cell death in response to cellular stress or DNA damage [56,57]. In this regard, BCL-2 and BAX are two essential downstream transcriptional targets involved in p53-dependent apoptosis. Moreover, the p53-mediated apoptotic response depends partially on the induction of NF-κB-p65 [55,58]. Therefore, we investigated the expression levels of BCL-2, BAX, p53, and NF-κB-p65 in LNCaP and HepG2 cells treated with BNHLE. Our results showed a concentration-dependent upregulation of p53 expression (Figure 6A,C) and downregulation of NF-kB-p65 (Figure 6B,D) in both cancer cells as compared to control cells. NF-κB-p65 promotes uncontrolled cell growth by reducing the apoptotic capacity of cancer cells [59,60], the reduced expression levels of NF-κB-p65 in both cancer cells might indicate the greater sensitivity of BNHLE to induce apoptosis in these cells. Moreover, recent research has shown that p53 is involved in cellular processes such as ROS generation, apoptosis, and cellular metabolism [61]. Therefore, the upregulation of p53 contributed to the induction of apoptosis via the generation of ROS. Additionally, the increased levels of p53 influenced directly BCL-2 expression, which plays a role in apoptosis regulation, as it is recognized from several studies that p53 may function by downregulating BCL-2 transcription in certain cancer cells [62,63]. Another study reported that the ethylacetate stem bark of *Boascia coriacea* (Pax.) promotes apoptosis in prostate cancer cells by decreasing the expression of *bcl-2* gene as compared to control cells [42]. Thus, the increased levels of p53 may result in the reduction of BCL-2 expression, leading to the activation of apoptotic pathways after BNHLE treatment.

**Figure 7:**
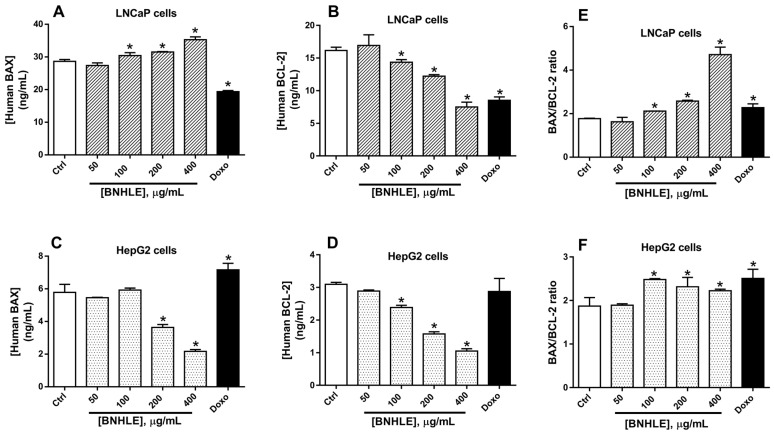
Expression levels of human BAX and BCL-2 in LNCaP (**A**,**B**) and HepG2 (**C**,**D**) as well as their respective BAX/BCL-2 ratio (**E**,**F**) after 24 h of treatment with *B. natalensis* hydroethanolic leaf extract (BNHLE). Cells were treated for 24 h with BNHLE, or doxorubicin hydrochloride (Doxo, 2 µM) or DMSO (0.5%) used as negative control (Ctrl). Equal amounts of proteins in cell lysates were used for the quantification of the expression levels of BAX and BCL-2 using commercially available BCL-2 Associated X protein and BCL-2 ELISA kits (Cat# EEL030 and Cat# BMS244-3, Thermo Fisher Scientific, Massachusetts, MA, USA), respectively. The BAX/BCL-2 ratio was calculated by dividing the BAX expression level against the BCL-2 level at each tested concentration. One-way ANOVA combined with Dunnett or Student–Newman–Keuls’s tests were used for data analysis. * means statistically significantly different (*p* < 0.05) vs. Ctrl.

To further understand this mechanistic pathway, we found that the human BAX expression levels increased significantly (*p* < 0.05) in LNCaP cells after exposure to BNHLE (100–400 µg/mL) (Figure 7A), while a dose-dependent decrease of BAX was observed in HepG2 cells after exposure to BNHLE (200–400 µg/mL) (Figure 7C). In contrast, the expression levels of BCL-2 were downregulated significantly (*p* < 0.05) in both cancer cells after treatment with BNHLE (100–400 µg/mL) in a dose-dependent manner (Figure 7B,D). It is known that BAX promotes apoptosis, while BCL-2 inhibits apoptosis and promotes cell survival [64], the results obtained in LNCaP cells indicating an increase of BAX expression and a decrease of BCL-2 expression, which corroborate with our previous data and further confirmed that apoptotic pathways are activated in these cells after treatment with BNHLE. Conversely, BAX and BCL-2 expression levels decreased in HepG2 cells, which might be misinterpreted as these data could not give a clear indication of apoptosis activation in these cells. Therefore, we resolved to determine the ratio between BAX and BCL-2, which is an indicator of a cell’s sensitivity to apoptosis that determines whether a cell will undergo apoptosis or survive. In general, a higher BAX/BCL-2 ratio means a higher susceptibility for apoptotic cell death [65].

As indicated in Figure 7E, a dose-dependent increase of BAX/BCL-2 ratio was observed in LNCaP cells, which clearly means the higher sensitivity of these cells to undergo apoptosis after treatment with BNHLE. In contrast, a moderate increase of BAX/BCL-2 ratio was observed in HepG2 cells after treatment with BNHLE (100–400 µg/mL) (Figure 7F), which is due to decreased levels of BAX in HepG2 cells after BNHLE treatment. However, this moderate increase of BAX/BCL-2 ratio compared to untreated control cells supports the activation of apoptosis in HepG2 cells. Overall, these data align with the above findings regarding the induction of apoptosis through caspase activation. Therefore, it can be suggested that phytochemicals within BNHLE induce LNCaP and HepG2 cancer cell death by promoting the activation of caspase-p53-BCL-2-dependent apoptosis.

### 2.5. Phytochemical Analysis of BNHLE Identifies Natural Compounds with Known Anticancer Property

Plant-derived phenolics and flavonoids have been demonstrated to inhibit cancer cell growth by interfering with diverse mechanistic pathways including the induction of cell cycle arrest and the activation of apoptosis [66,67,68]. Due to the importance of these two groups of phytochemicals in cancer therapy, we decided to determine their concentrations in *B. natalensis* hydroethanolic leaf extract (BNHLE). As presented in Table 3, the phytochemical analysis of BNHLE showed the presence of flavonoids (24.45 mgQE/g extract) and phenolics (84.64 mgGAE/g extract). *B. natalensis* methanolic leaf extract (BNMLE) was richer in these phytochemicals with flavonoids (50.88 mgQE/g extract) and phenolics (95.90 mgGAE/g extract). However, the anticancer potential of both extracts was quite similar, and BNHLE was considered in future studies based on its selective cytotoxic effect compared to BNMLE.

**Figure 8:**
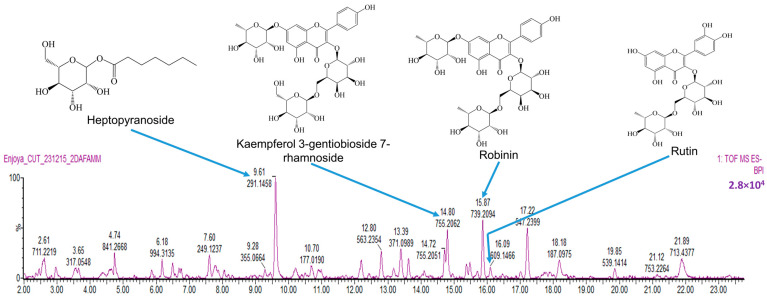
Liquid chromatography-mass spectrometric (LC-MS) profile of *B. natalensis* hydroethanolic leaf extract (BNHLE). Twenty microliters of BNHLE were injected to Waters Cyclic Quadrupole time-of-flight (qTOF) mass spectrometer (MS) connected to a Waters Acquity ultra-performance liquid chromatograph (UPLC) (Waters, Milford, MA, USA) for high-resolution UPLC-MS analysis. Column eluate first passed through a Photodiode Array (PDA) detector before going to the mass spectrometer, allowing simultaneous collection of UV and MS spectra. Compounds within the extract were tentatively identified using MSDIAL and MSFINDER databases.

We further analysed the LC-MS profile of BNHLE, which led to the identification of several compounds among which heptopyranoside (R_t_: 9.61 min), kaempferol 3-gentiobioside 7-rhamnoside (R_t_: 14.80 min), robinin (R_t_: 15.87 min), and rutin (R_t_: 16.09 min) (Figure 8, Table 4). Robinin (537 mg/L) has been reported to inhibit pancreatic cancer cell proliferation and migration [69]. However, no research was found on its anticancer property on prostate and liver cancer cells. In contrast, it was reported that rutin (141 mg/L) induced apoptosis in many cancer cell lines, including human prostate cancer cells (DU-145, PC-3, and LNCaP) [70] and hepatocellular carcinoma (HepG2) cells [71], which occurred via a decrease of BCL-2 and an increase of p53 protein expression [70,72]. Based on these data, the antiproliferative effect of BNHLE may be attributed to the presence of rutin and robinin. Several unknown compounds that could not be identified by LC-MS method may also be involved in the anticancer property. For instance, other studies reported the presence of over 200 triterpenoidal alkaloids from various plants of the genus Buxus [27,28], and some of these alkaloids exhibited a promising antiproliferative effect against several cancer cells, including melanoma, colorectal carcinoma, hepatocellular carcinoma, prostate cancer cells, and breast cancer cells, by inducing cell cycle arrest, apoptosis, and autophagy [29,30,31].

To date, 12 triterpenoidal alkaloids, namely *O*^2^-natafuranamine, *O*^10^-natafuranamine, cyclonataminol, buxaminol A, buxamine A, buxaminol C, 31-demethylbuxaminol A, buxafuranamide, buxalongifolamidine, cyclobuxophylline K, methyl syringate, and *p*-coumaroylputrescine, were isolated from the methanolic extract of *B. natalensis* [35], but no research on their anticancer property is yet reported. Furthermore, identifying the mechanisms through which these potential compounds affect different signaling pathways may unravel the identification of appropriate targets and speed up the development of safe and effective drugs against hepatocellular and prostate cancers using *B. natalensis* as a valuable botanical source. Thus, *B. natalensis* can be considered as a potential source of bioactive compounds against hepatocellular carcinoma and prostate cancers, and a bioassay-guided fractionation of its hydroethanolic extract (BNHLE) will be conducted to fully identify and characterize anticancer compounds from this plant species.

## 3. Materials and Methods

### 3.1. Plant Material and Extraction

Fresh leaves of *B. natalensis* were collected at the University of Pretoria Botanical Garden, Pretoria (South Africa). Herbarium specimen was prepared, and identification was made by Ms. Magda Nel and Mrs. Elsa van Wyk of the HGWJ Schweickerdt Herbarium (PRU), University of Pretoria, with the identification number PRU 119044. The fresh leaves were dried in a well-ventilated room at an ambient temperature, and the dried leaves were grounded into a fine powder. Leaf powder (50 g) was extracted separately with 500 mL of ethanol/water (80:20) or methanol at room temperature by maceration with continuous shaking for 48 h. After that, each mixture was filtered through Whatman No.1 filter paper into pre-weighed beakers, and the filtrates obtained were concentrated to dryness under a stream of cold air to obtain a residue that constituted the crude extracts. The aqueous extraction was also done after boiling for 1 h the mixture of 50 g of leaf powder in 500 mL of distilled water. After filtration with sterile cotton followed with Whatman No.1 filter paper, the aqueous extract was obtained after drying the filtrate in a freeze dryer. After drying, the beakers were reweighed to determine quantity extracted, and the percentage yield of extraction was calculated (Table 3). The dried extracts were stored in a cold room (4 °C) until use.

### 3.2. Cell Proliferation Assay

#### 3.2.1. Cell Culture

Eight cancer cell lines, namely, human breast carcinoma cells (MCF-7), 4T1 mammary carcinoma cells, human colorectal carcinoma cells (Caco-2), human cervical carcinoma cells (HeLa), human lung carcinoma cells (A549), human hepatocellular carcinoma (HepG2), DU145 (androgen-insensitive), and LNCaP (androgen-sensitive) human prostate cancer cell lines, were obtained from the American Type Culture Collection (ATCC) (Rockville, MD, USA) or the European Collection of Authenticated Cell Cultures (ECACC) (Porton Down, UK). MCF-7, Caco-2, A549, HeLa, and HepG2 were grown in Dulbecco’s Modified Eagle’s Medium (DMEM) high glucose (4.5 g/L) containing 4 mM L-glutamine and sodium pyruvate (Cytiva Hyclone, Logan, UT, USA) while DU145 cells were grown DMEM/nutrient mixture F-12 containing 2.5 mM L-glutamine and 15 mM HEPES buffer (Cytiva Hyclone, Logan, UT, USA). LNCaP and 4T1 were grown in Roswell Park Memorial Institute (RPMI) 1640 medium (Cytiva Hyclone, Logan, UT, USA). Chang liver cells (obtained from ECACC), which is a normal cell line, were cultured in Minimal Essential Medium (MEM) containing Earle’s Balanced Salt Solution (EBSS) and L-Glutamine (2.0 mM) (Cytiva Hyclone, Logan, UT, USA). All culture media were supplemented with 10% (*v*/*v*) fetal bovine serum (FBS) (Cytiva Hyclone, Logan, UT, USA) and 1% penicillin/streptomycin (P/S) (Cytiva Hyclone, Logan, UT, USA). However, P/S was only used during cell culture, and culture media without P/S was during experiments as antibiotics in culture media can interfere with anticancer drug testing by altering cell behavior and potentially impacting the accuracy of anticancer drug efficacy assessments. The cells were maintained in a CO_2_ incubator (Nϋve, Ankara, Turkey) under standard cell-culture conditions (37 °C in a humidified environment with 95% air and 5% CO_2_). At 70–80% confluency, the cells were trypsinised with 0.25% trypsin/EDTA (Cytiva Hyclone, Logan, UT, USA) and split at a ratio of 1:5 for further passaging. The cell viability was checked with trypan blue 0.4% (Cytiva Hyclone, Logan, UT, USA) on an automat cell counter (NanoEntek, Seoul, Republic of Korea), and only cell suspensions with a cell viability greater than 90% were used for subsequent cell-based assays.

#### 3.2.2. Cytotoxicity Assay

Each cell line was seeded at a density of 1 × 10^4^ cells per well on 96-well microtiter plates, and the plates were incubated overnight under standard cell-culture conditions to allow cell attachment. After incubation, the cells were treated with different leaf extracts (15.625–500 µg/mL), as well as doxorubicin (0.1–50 µM) dissolved in dimethyl sulfoxide (DMSO) and further diluted in fresh culture medium. In each experiment, the concentration of DMSO in the medium did not exceed 0.5% (negative control). The plates were further incubated for 48 h under standard cell-culture conditions. Thereafter, the culture medium containing tested samples was discarded and replaced by fresh medium (200 µL) with 30 µL of thiazolyl blue tetrazolium bromide (5 mg/mL) (Melford, Ipswich, UK) dissolved in phosphate-buffered saline (PBS). After 4 h of incubation under standard cell-culture conditions, the culture medium was gently aspirated, and the formazan crystals were dissolved in 50 µL of DMSO and kept in the dark for 15 min at room temperature. The absorbance was measured spectrophotometrically at 570 nm on a SpectraMax iD3 multi-mode microplate reader (Molecular Devices, San Jose, CA, USA) after shaking for 2 min.

#### 3.2.3. Determination of IC_50_ Values and Selectivity Indexes

The cell viability was calculated as the percentage of negative control (cells treated with 0.5% DMSO) (Formula (1)). The percentage of cell viability serves as a quantitative indicator of cellular metabolic activity of live cells, and lower cell viability indicates greater potency of tested samples.

Cell viability (%) = [(A_0_ − A_1_)/(A_2_ − A_1_)] × 100 (1)

A_0_ is absorbance of sample, A_1_ is absorbance of blank, and A_2_ is absorbance of negative control (0.5%DMSO).

The 50% inhibitory concentrations (IC_50_) for cancer cells and normal cells were determined with GraphPad Prism 6.0 software (GraphPad Software, Inc., San Diego, CA, USA) by plotting the non-linear regression curve of percentage of cell viability versus the logarithm (log10) of concentrations of each sample. The selectivity index (SI) values, which reflect the differential cytotoxicity of a tested sample between normal and cancer cells, were calculated by dividing the IC_50_ of normal cells against the IC_50_ of each cancer cell type in the same units (Formula (2)). The higher the SI value of a tested sample, the more selective it is. An SI > 1 indicates a higher toxic effect against cancer cells than against normal cells, while an SI < 1 indicates the general toxicity against both normal and cancer cells.

Selectivity Index (SI) = IC_50_ (normal cells)/IC_50_ (cancer cells) (2)

### 3.3. Colony-Formation Assay

The most sensitive cancer cells that showed the lowest IC_50_ values were selected for this experiment. These cells were seeded in six-well plates at a density of 1000 cells per well and then treated for 24 h with different concentrations (50, 100, 200, and 400 µg/mL) of the most active extract or doxorubicin hydrochloride (2 µM), or DMSO (0.5%) used as control. Culture media were changed every 3 days. After 15 days, the cells were washed with phosphate-buffered saline (PBS) and fixed with ice-cold methanol at 25 °C for 15 min. Fixed cells were then stained with 0.5% crystal violet (Sigma Aldrich, Burlington, MA, USA) for 30 min, followed by washing with water to remove unbound dye. Thereafter, the plates were air-dried, and images of colonies in the wells were captured. Moreover, crystal violet fixed by colonies was dissolved with a solution (33% acetic acid), and the absorbance was measured at 550 nm on a SpectraMax iD3 multi-mode microplate reader (Molecular Devices, San Jose, CA, USA).

### 3.4. Assessment of Intracellular Reactive Oxygen Species Production

Selected cancer cells (1 × 10^4^/well) were seeded in a 96-well black/clear bottom microtiter plate and allowed to attach overnight. These cells were treated for 45 min with 100 µL of 20 µM of the fluorescent probe 2′,7′-dichlorodihydrofluorescein diacetate (DCFH-DA) diluted in culture medium containing 2% FBS. After that, DCFH-DA solution was discarded and replaced by 200 µL of fresh culture medium containing different concentrations (100 and 200 µg/mL) of *B. natalensis* hydroethanolic leaf extract (BNHLE), followed by 24 h of incubation under standard culture conditions. DMSO (0.5%) was used as negative control, while 50 µM H_2_O_2_ (Sigma-Aldrich, Steinheim, Germany) was used as positive control. Images were captured using a Flexacam C1 camera (Leica Microsystems GmbH, Wetzlar, Germany) connected to a fluorescence microscope (Leica Microsystems GmbH, Wetzlar, Germany) using an excitation/emission filter (480/535 nm) on 20× objective. The cell fluorescence was measured at the wavelengths of 485 nm (excitation) and 535 nm (emission) on a SpectraMax iD3 multi-mode microplate reader (Molecular Devices, San Jose, CA, USA), and intracellular ROS levels were expressed as a percentage of negative control cells.

### 3.5. Assessment of the Caspase Activation in Cancer Cells

The most sensitive cancer cells were seeded at a density of 1 × 10^4^/well in a 96-well black/clear bottom microtiter plate, and these cells were treated at different concentrations of BNHLE (100 and 200 µg/mL). Doxorubicin hydrochloride (2 µM) was used as the positive control while DMSO (0.5%) was used as the negative control. The plates were incubated for 18 h at 37 °C under standard culture conditions. The caspase -3, -7, and -9 activities were monitored with the Caspase-Glo^®^-3/-7 assay kit (Cat# G8090, Promega, Madison, WI, USA) and Caspase-Glo^®^-9 assay kit (Cat# G8210, Promega, Madison, WI, USA) according to the manufacturer’s guidelines. In fact, after preparing the reagent, 100 µL of each reagent was added per well and incubated for 1 h at room temperature in the dark. After this incubation, the luminescence was measured on a SpectraMax iD3 multi-mode microplate reader (Molecular Devices, San Jose, CA, USA). The data obtained were expressed as fold change of cells treated with 0.5% DMSO (negative control).

### 3.6. Quantification of the Expression of Human BAX, BCL-2, p53, and NF-κB-p65 in Cancer Cells

The Enzyme-Linked Immunosorbent Assay (ELISA) technique was applied to determine the expression levels of human B-cell lymphoma 2 protein (BCL-2), BCL-2-associated X protein (BAX), tumor suppressor protein p53, and nuclear factor-kappa-B p65 subunit (NF-κB-p65) after treatment of cancer cells with BNHLE. In fact, the most sensitive cancer cells (500,000 cells/well) were seeded into six-well plates and were allowed to attach overnight under standard cell-culture conditions. These cells were treated with different concentrations of BNHLE, 0.5% DMSO (negative control), or doxorubicin (2 µM). The plates were incubated for 24 h under standard cell-culture conditions. After that, cells were washed twice with phosphate-buffered saline (PBS), and the cell lysates were obtained in M-PER™ Mammalian Protein Extraction Reagent containing EDTA-free Pierce Protease and Phosphatase Inhibitor Tablets (Thermo Fisher Scientific, Lenexa, KS, USA). The cell homogenates were centrifuged at 10,000 rpm at 4 °C, and aliquots of cell supernatants were kept at −20 °C. After determining the quantity of protein in each cell lysate with Pierce^TM^ BCA protein assay kit (Thermo Fisher Scientific, Lenexa, KS, USA), equal amount of proteins from each cell lysate was used for the quantification of the expression levels of human BCL-2, BAX, p53, and NF-κB-p65 using the human BCL-2 and BCL-2 Associated X protein ELISA kits (Cat# EEL030 and Cat# BMS244-3, Thermo Fisher Scientific, Massachusetts, MA, USA); and human p53 and NF-κB-p65 ELISA kits (Cat# E-EL-H1388 and Cat# E-EL-H0910, Elabscience Biotechnology Inc., Houston, TX, USA) according to the manufacturer’s instructions, respectively.

### 3.7. Phytochemical Analysis of B. natalensis Hydroethanolic Leaf Extract (BNHLE)

The total flavonoid and phenolic contents were measured in the extracts using, respectively, the Folin–Ciocalteu and aluminum chloride colorimetric methods reported in previous publications [73,74]. Upon completion of these experiments, the results were converted into equivalents of milligrams of gallic acid (mgGAE per g of dry extract) and quercetin (mgQE per g of dry extract) for the total phenolic and flavonoid contents, respectively.

A tentative identification of compounds within the most prominent extract (BNHLE) was carried out using liquid chromatography mass spectrometry (LC-MS). A Waters Cyclic Quadrupole time-of-flight (qTOF) mass spectrometer (MS) connected to a Waters Acquity ultra-performance liquid chromatograph (UPLC) (Waters, Milford, MA, USA) was used to perform a high-resolution UPLC-MS analysis. Separation was achieved on a Walters HSS T3 (Waters Corporation, Milford, MA, USA) with a dimension of 2.1 × 150 mm and a particle size of 1.8 µm. The extract (40 mg) was dissolved in 2 mL methanol and passed through a 0.22 µm filter. The filtrate was further diluted 10-fold in a mixture of acetonitrile and water (1:1 *v*/*v*), and 20 μL was injected for analysis. The mobile phase was made of two solvent systems: solvent A consisted of 0.1% formic acid while solvent B was made of acetonitrile containing 0.1% formic acid. The gradient started at 100% of A for 1 min and changed to 72% of A with 28% of B over 22 min in a linear way. It then went to 40% of B over 50 s and a wash step of 1.5 min at 100% of B, followed by re-equilibration to initial conditions for 4 min. The flow rate was 0.25 mL/min, and the column temperature was maintained at 55 °C. The signals of the separated compounds were recorded at 254 nm. Based on the accurate mass elemental compositions, the compounds within the extract were tentatively identified using MSDIAL and MSFINDER (RIKEN Center for Sustainable Resource Science: Metabolome Informatics Research Team, Kanagawa, Japan) [75,76]. According to the spectral match between the in-silico and measured spectra, a score (out of 10) is assigned to each of the possible compound matches with the highest score being accepted as the most likely (assuming a score of at least 4). Additionally, the identified compounds were semi-quantified (mg/L) in a relative manner against a calibration curve established by injecting a range of 3-caffeoylquinic acid (3CQA) standard.

### 3.8. Statistical Analysis

All experiments were carried out in triplicate (n = 3). and the results are presented as mean ± SEM (standard error of mean) values. Statistical analysis was carried out with GraphPad Prism 6.0 software (GraphPad Software, Inc., San Diego, CA, USA). The comparison of data among tested samples and/or positive controls was done using one-way analysis of variance (ANOVA) and Student–Newman–Keuls or Dunnett’s tests in GraphPad Prism 6.0 software. Results were considered significantly different when the *p* value was greater than 0.05.

## 4. Conclusions

The present study demonstrated that *B. natalensis* hydroethanolic extract (BNHLE) selectively kills hepatocellular carcinoma (HepG2) and prostate (LNCaP) cancer cells, with minimal cytotoxic effect on normal Chang liver cells. The high selectivity of BNHLE can be associated with the presence of phytochemicals, which caused significant damage in both cancer cells while preserving the normal cells, supporting its strong antiproliferative effect and favourable safety profile. The antiproliferative effect of BNHLE is mostly attributed to the modulation of key molecular pathways, including the stimulation of ROS generation and caspase -3/-7, and -9 activities in both cancer cell lines. Additionally, BNHLE promotes apoptosis in both cancer cell lines by increasing the expression levels of p53, while suppressing the expression of NF-κB-p65 and BCL-2 protein levels. Moreover, an increase of BAX/BCL-2 ratio in both cancer cells clearly supported the fact that these cells undergo apoptosis after BNHLE treatment. The presence of bioactive phytochemicals in BNHLE highlights its therapeutic potential and prompts the need to carry out comprehensive investigations to isolate and characterize lead compounds for the development of anticancer therapies. Besides, given the limitations of doxorubicin therapy on LNCaP and HepG2 cells versus Chang liver cells, using BNHLE is a good and alternative way to lessen adverse effects of traditional chemotherapy medicines. At the current stage, BNHLE can be considered as a potential source of anticancer agents, and to fully validate its therapeutical potential, further empirical research is necessary using in vivo models to support the results obtained in the current study.

## Figures and Tables

**Table 1:** Inhibitory concentrations (IC_50_) values of different *B. natalensis* leaf extracts against different cancerous and non-cancerous cell lines.

IC_50_ (µg/mL) of Plant Extracts									
*Buxus natalensis*	Cancer Cell Lines								Normal Cell
	Lung Cancer	Cervical Cancer	Liver Cancer	Breast Cancer		Prostate Cancer		Colorectal Cancer	Chang Liver
	A549	HeLa	HepG2	4T1	MCF-7	DU145	LNCaP	Caco-2	
Hydroethanolic	>500	378.90 ± 3.16 ^a^	78.01 ± 1.30 ^a^	105.20 ± 6.40 ^a^	>500	258.30 ± 2.47 ^a^	47.39 ± 1.98 ^a^	>500	329.70 ± 2.62 ^a^
Aqueous	>500	>500	>500	>500	>500	>500	>500	>500	>500
Methanolic	>500	362.20 ± 5.69 ^a^	79.07 ± 1.42 ^a^	98.00 ± 2.89 ^a^	>500	219.33 ± 2.40 ^b^	49.18 ± 2.75 ^a^	>500	205.50 ± 9.58 ^b^
Doxorubucin (µM)	1.02 ± 0.50	1.00 ± 0.52	4.21 ± 1.09	1.57 ± 0.25	1.96 ± 0.28	0.92 ± 0.26	1.73 ± 0.12	57.33 ± 4.15	1.22 ± 0.13

Data are presented as means of triplicate measurements ± standard error of mean; Values with different letters are significantly different at *p* < 0.05. IC_50_: concentration required to inhibit the cell growth by 50% compared to negative control (DMSO 0.5%). All experiments were carried out in triplicate (n = 3), and the results are presented as mean ± SEM (standard error of mean) values.

**Table 2:** Selectivity index (SI) values of plant extracts as compared to Chang liver cell line.

*Buxus natalensis*	SI Values							
	A549	HeLa	HepG2	4T1	MCF-7	DU145	LNCaP	Caco-2
Hydroethanolic	nd	0.87	4.22	3.13	nd	1.28	6.96	nd
Aqueous	nd	nd	nd	nd	nd	nd	nd	nd
Methanolic	nd	0.57	2.59	2.10	nd	0.94	4.17	nd
Doxorubucin	1.19	1.22	0.29	0.78	0.62	1.33	0.71	0.02

nd = not determined. The higher the SI value of an extract, the more selective it is. An SI > 1 indicates higher efficacy against cancer cells than normal cells, while an SI < 1 indicates the general toxicity against both normal and cancer cells.

**Table 3:** Percentage yield of extraction and phytochemical contents of *B. natalensis* leaf extracts.

*Buxus natalensis*	% Yield of Extraction (g Extract/100 g Dry Material)	Phenolic Content (mgGAE/g Extract)	Flavonoid Content (mgQE/g Extract)
Hydroethanolic	15.78	84.64 ± 1.65	24.45 ± 1.35
Aqueous	33.55	68.42 ± 1.53	16.84 ± 2.26
Methanolic	13.32	95.90 ± 2.10	50.88 ± 3.99

**Table 4:** Tentative assignment of compounds in *B. natalensis* hydroethanolic leaf extract using LC-MS in negative mode ionization.

Peak N°	R_t_ (min)	[M − H]^−^ (*m*/*z*)	Tentative Assignment (Compound Name)	Ontology	Molecular Formula	Total Score	Peak Height Intensity	Conc. in Extract vs. 3CQA (mg/L)
1	6.18	971.31	UNPD33759	Oligosaccharides	C_72_H_120_O_60_	4.35	6921	256
2	9.61	291.14	Heptopyranoside	Fatty acyl glycosides of mono- and disaccharides	C_13_H_24_O_7_	4.57	25,627	947
3	13.18	431.19	MINEs-320976	Saccharolipids	C_20_H_32_O_10_	5.33	2471	91
4	14.51	625.14	Quercetin 3-glucosyl-(1->2)-galactoside	Flavonoid-3-O-glycosides	C_27_H_29_O_17_	7.22	1307	48
5	14.80	755.20	Kaempferol 3-gentiobioside 7-rhamnoside	Flavonoid-7-O-glycosides	C_33_H_40_O_20_	6.75	19,443	719
6	15.37	609.14	Kaempferol derivative	Flavonoid-3-O-glycosides	C_27_H_30_O_16_	6.75	3585	133
7	15.47	581.22	(7′R)-(+)-Lyoniresinol 9′-glucoside	Lignan glycosides	C_28_H_38_O_13_	6.26	3964	147
8	15.87	739.20	Robinin	Flavonoid-7-O-glycosides	C_33_H_40_O_19_	6.64	14,525	537
9	16.09	609.14	Rutin	Flavonoid-3-O-glycosides	C_27_H_30_O_16_	8.09	3813	141
10	16.46	463.08	Quercetin 3-galactoside	Flavonoid-3-O-glycosides	C_21_H_20_O_12_	7.90	754	28
11	17.22	547.23	UNPD111558	Terpene glycosides	C_25_H_40_O_13_	6.90	12,752	471
12	17.72	593.15	Astragalin 7-rhamnoside	Flavonoid-7-O-glycosides	C_27_H_30_O_15_	8.12	1428	53
13	17.75	457.07	Unknown	-	-	-	719	27
14	18.08	597.24	Unknown	-	-	-	434	16
15	18.11	447.09	Astragalin	Flavonoid-7-O-glycosides	C_21_H_20_O_11_	8.09	499	18

R_t_: retention time; 3CQA: 3-Caffeoylquinic acid.

## Data Availability

The datasets used and/or analyzed during the current study are available from the corresponding authors on reasonable request.

## Supplementary Materials

The following supporting information can be downloaded at: https://www.mdpi.com/article/10.3390/ijms26094173/s1.

## Abbreviations

The following abbreviations are used in this manuscript:

ROS	Reactive Oxygen Species
p53	Tumor suppressor protein 53
BCL-2	B-cell lymphoma 2
BAX	BCL-2-associated X protein
NF-κB-p65	Nuclear factor-kappa B-p65 subunit
BNHLE	*Buxus natalensis* hydroethanolic leaf extract
BNMLE	*Buxus natalensis* methanolic leaf extract
BNALE	*Buxus natalensis* aqueous leaf extract
LC-MS	Liquid chromatography mass spectrometry
