# Supplementary material for: Buxus natalensis (Oliv.) Hutch (Buxaceae) Exhibits Its Anticancer Potential by Stimulating ROS Production and Caspase-p53-BCL-2-Dependent Apoptosis in Hepatocellular Carcinoma and Prostate Cancer Cell Lines
Source: Int J Mol Sci. 2025 Apr 28;26(9):4173. doi: 10.3390/ijms26094173 (PMC12071716; doi:10.3390/ijms26094173)
Supplement: Supplementary file 1 [file ijms-26-04173-s001.zip › ijms-3589817-supplementary.pdf]

Supplementary material

Buxus natalensis (Oliv.) Hutch (Buxaceae) Exhibits Its Anticancer Potential by Stimulating ROS Production and Caspase-p53-BCL-2-Dependent Apoptosis in Hepatocellular Carcinoma and Prostate Cancer Cell Lines

Emmanuel Mfotie Njoya *, Gaetan T. Tabakam, Chika I. Chukwuma and Tshepiso J. Makhafola *

Centre for Quality of Health and Living, Faculty of Health and Environmental Sciences, Central University of Technology, Bloemfontein 9301, Free State, South Africa; tgaetan@cut.ac.za (G.T.T.); cchukwuma@cut.ac.za (C.I.C.)
* Correspondence: enjoya@cut.ac.za (E.M.N.); jmakhafola@cut.ac.za (T.J.M.)

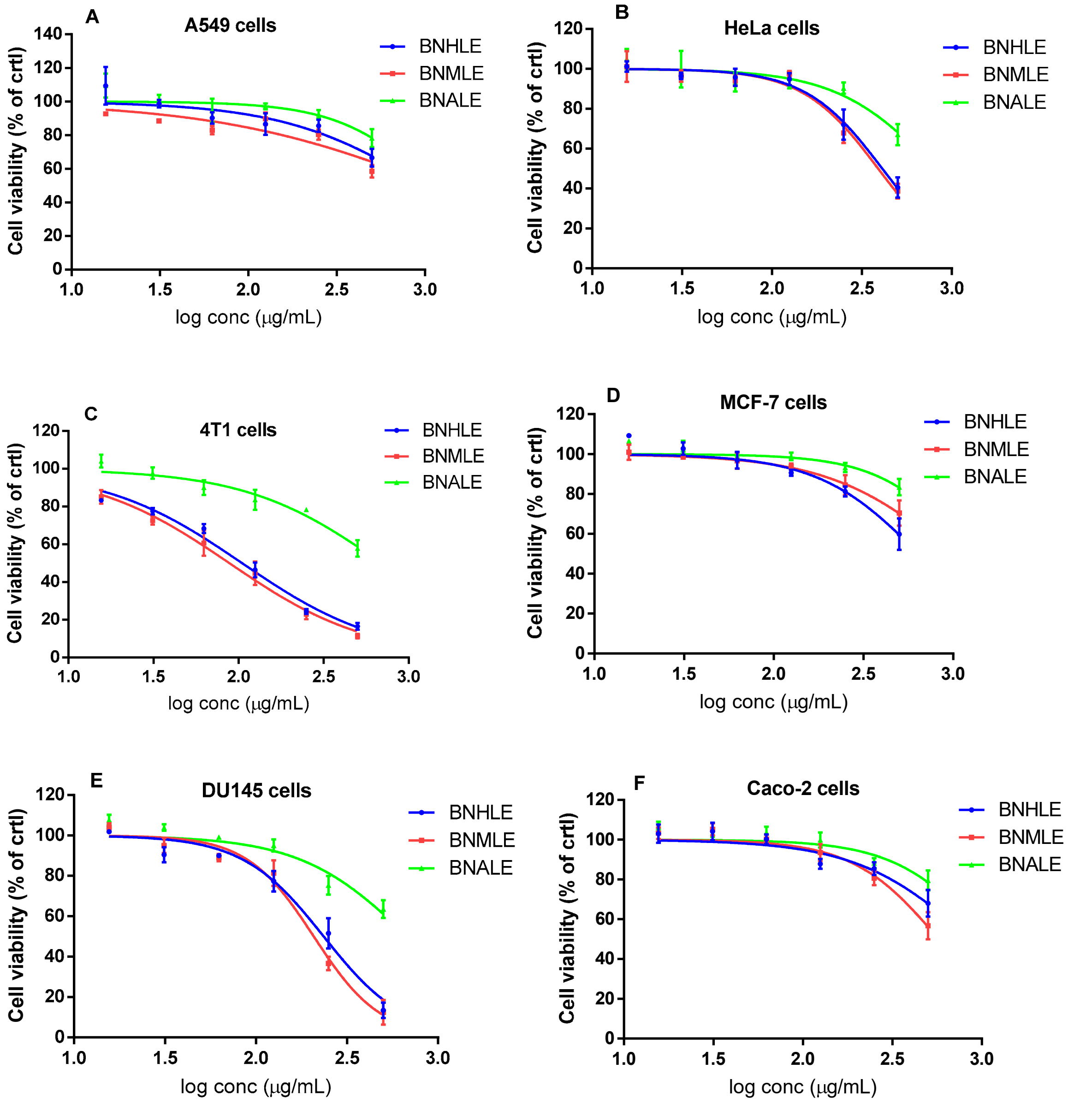

Figure S1: Cytotoxic effect of *Buxus natalensis* leaf extracts on A549 (**A**), HeLa (**B**), 4T1 (**C**), MCF-7 (**D**), DU145 (**E**), and Caco-2 (**F**) cell lines. Ten thousand cells were seeded per well for each cell line on 96-

well microtiter plates, and the cells were treated for 48 h with different leaf extracts (15.625 - 500μg/mL) under standard cell-culture conditions. The cell viability was estimated as a percentage of cells treated with DMSO (0.5%) considered as 100%. *B. natalensis* hydroethanolic leaf extract (BNHLE); *B. natalensis* methanolic extract (BNMLE); *B. natalensis* aqueous leaf extract (BNALE).
